# Deficient EBV-Specific B- and T-Cell Response in Patients with Chronic Fatigue Syndrome

**DOI:** 10.1371/journal.pone.0085387

**Published:** 2014-01-15

**Authors:** Madlen Loebel, Kristin Strohschein, Carolin Giannini, Uwe Koelsch, Sandra Bauer, Cornelia Doebis, Sybill Thomas, Nadine Unterwalder, Volker von Baehr, Petra Reinke, Michael Knops, Leif G. Hanitsch, Christian Meisel, Hans-Dieter Volk, Carmen Scheibenbogen

**Affiliations:** 1 Institute for Medical Immunology, Charité University Medicine Berlin, Campus Virchow, Berlin, Germany; 2 Julius Wolff Institute, Charité University Medicine Berlin, Campus Virchow, Berlin, Germany; 3 Labor Berlin GmbH, Immunology Department, Charité University Medicine Berlin, Campus Virchow, Berlin, Germany; 4 Institute for Medical Diagnostics, Berlin, Germany; 5 Berlin-Brandenburg Center for Regenerative Therapies (BCRT), Charité University Medicine Berlin, Germany; 6 Department Nephrology, Charité University Medicine Berlin, Germany; University of British Columbia, Canada

## Abstract

Epstein-Barr virus (EBV) has long been discussed as a possible cause or trigger of Chronic Fatigue Syndrome (CFS). In a subset of patients the disease starts with infectious mononucleosis and both enhanced and diminished EBV-specific antibody titers have been reported. In this study, we comprehensively analyzed the EBV-specific memory B- and T-cell response in patients with CFS. While we observed no difference in viral capsid antigen (VCA)-IgG antibodies, EBV nuclear antigen (EBNA)-IgG titers were low or absent in 10% of CFS patients. Remarkably, when analyzing the EBV-specific memory B-cell reservoir *in vitro* a diminished or absent number of EBNA-1- and VCA-antibody secreting cells was found in up to 76% of patients. Moreover, the *ex vivo* EBV-induced secretion of TNF-α and IFN-γ was significantly lower in patients. Multicolor flow cytometry revealed that the frequencies of EBNA-1-specific triple TNF-α/IFN-γ/IL-2 producing CD4^+^ and CD8^+^ T-cell subsets were significantly diminished whereas no difference could be detected for HCMV-specific T-cell responses. When comparing EBV load in blood immune cells, we found more frequently EBER-DNA but not BZLF-1 RNA in CFS patients compared to healthy controls suggesting more frequent latent replication. Taken together, our findings give evidence for a deficient EBV-specific B- and T-cell memory response in CFS patients and suggest an impaired ability to control early steps of EBV reactivation. In addition the diminished EBV response might be suitable to develop diagnostic marker in CFS.

## Introduction

Chronic Fatigue Syndrome (CFS) is characterized by severe fatigue with typical post-exertional delay to recover from exhaustion, cognitive dysfunctions and flu-like symptoms [1], [2]. CFS is diagnosed based on clinical Center of Disease Control criteria scores known as Fukuda criteria [3] or on the Canadian Consensus Definition from 2004 [1]. Diagnosis of CFS is often restrained as many symptoms are not disease-specific and no diagnostic test could be established for CFS so far [4], [5], [6], [7].

Hallmarks of CFS are immune dysregulation and immune activation [8], [9], [10]. Diminished natural killer (NK)-cell cytotoxicity and reduced NK-cell derived perforin have been repeatedly reported for CFS patients [8], [10], [11]. Furthermore, increased frequencies of activated HLA-DR class II-positive CD8^+^ T cells were proposed as immunological activation markers in CFS [10], [12], [13]. Straus *et al.* showed reduced proliferative responses of lymphocytes and reduced frequencies of CD4^+^ T cells [14]. Similarly Curriu *et al.* reported diminished proliferation of T cells but enhanced frequencies of regulatory T cells [15]. Broderick and colleagues described a dysregulation of Th-17 priming by enhanced levels of IL-13, IL-2 and IL-8 but decreased levels of IL-5 and IL-23 in post-infectious CFS patients [16], [17]. Furthermore, the group of Skowera *et al.* reported an effector memory cell responsiveness bias towards type 2 in patients with CFS [12].

CFS onset typically goes along with a viral illness. Various viruses have been reported to trigger CFS. In 2009, it was published that the retrovirus XMRV is linked to CFS. Although this turned out to be a laboratory contamination, it called attention to this so far neglected disease [18], [19], [20], [21]. Herpes viruses as cause of CFS have been discussed for decades. However, stringent evidence for a clear association of enhanced or altered viral load and disease is still lacking [22], [23], [24], [25], [26], [27]. Further, in CFS data about altered serological responses against viruses of the herpes group are not consistent. Several groups reported more frequent detection of HHV6/7 load and elevated antibody titers [27], [28], [29], [30], [31] a finding that was not confirmed by others [32], [33]. Increased IgG to human cytomegalovirus (CMV), EBV viral capsid antigen (VCA), HHV-6, Herpes-Simplex Virus (HSV)-1, HSV-2 and Coxsackie viruses were reported in CFS in some studies [34], [35], [36], but not in others [37], [38].

Numerous studies have tried to find evidence for an association of CFS with EBV. In a subset of patients, CFS begins with infectious mononucleosis and enhanced EBV-specific antibody titers have been reported. Lerner *et al.* found serum IgM antibodies to EBV-VCA in CFS patients but not in controls and recently reported elevated antibodies against EBV-dUTPase and EBV-DNA polymerase in a subset of CFS patients [39], [40]. Consistent with these data, elevated titers of early antigen (EA)-IgG and antibodies to ZEBRA, a product of the immediate early EBV gene BamHI Z fragment leftward open reading frame (BZLF)-1, were detected in CFS patients [31], [41]. No differences in IgG titers against EBV-VCA, EBV nuclear antigen (EBNA)-1 and EA were reported in other studies [37], [42], [43].

The orally transmitted EBV initially targets the mucosal epithelium and remains in a life-long latency in memory B cells [44], [45], [46]. In healthy subjects the EBV genome usually remains latent in the so-called latency phase 0 and EBV replication is latent and without production of infectious virions [47], [48]. [49], [50]. This latency is controlled by NK- and T-cell responses. Replication occurs in different cycles, including latency I characterized by the expression of EBNA-1, latency II characterized by latent membrane proteins (LMP)-1 and LMP-2, and latency III when EBNA-2, -3 and -6 are also expressed [51], [52]. During lytic reactivation the EBV immediate-early genes BZLF-1 and BRLF-1 are expressed. These genes activate viral and cellular promoters that induce early, lytic and late viral gene expression and high amplification of the EBV genome [53]. EBER genes encode for regulatory RNAs. EBER-DNA can be used as a sensitive tool for the detection of EBV-infected cells, and the EBER-DNA copy number is related to the copy number of EBV-DNA molecules [54], [55]. No clear differences in EBV-DNA levels in blood and gastro-intestinal biopsies of CFS patients could be demonstrated yet [33], [37], [42].

At the Charité, we take care of patients with CFS in our outpatient clinic for adult immunodeficiencies as a subset of our CFS patients have concomitant immunoglobulin deficiency. Our observation of both elevated VCA-IgM and lack of EBNA-IgG in a subset of patients with CFS prompted us to perform a comprehensive analysis of the EBV-specific immune response. By comparing memory B- and T-cell responses of CFS patients with healthy EBV-infected subjects, we observed a profound deficiency in EBV-specific B- and T-cell memory response in the majority of CFS patients resembling the deficiency of EBV memory responses described in autoimmune diseases [56] and chronic HIV infection [57], [58], [59].

## Materials and Methods

### Ethics statement

The study was approved by the institutional ethics committee – Charité Universitätsmedizin Berlin and written informed consent was provided by all subjects. Only adults were included.

### Study population and specimen collection

Patients were diagnosed with CFS according to Fukuda criteria at our outpatient clinic between 2007 and 2013 [3]. Patients with other medical or neurological diseases were excluded. Patients who had a concomitant immunoglobulin deficiency were excluded when they fulfilled the diagnostic criteria for CVID or required immunoglobulin substitution due to recurrent bacterial infections. Two consecutive cohorts of patients (Table 1) were analyzed for EBV antibodies. We excluded 1/64 seronegative patients (1.6%) and 4/61 controls (7%) from our analyses in cohort 1 and 28/411 (6.8%) patients in cohort 2. Due to similar numbers of seronegative patients and controls, the interpretation of our data is not affected. A subset of patients from cohort 1 was analyzed for B-cell memory response by ELISpot, and EBV-induced T-cell cytokines. A subset of patients from cohort 2 was analyzed for EBV viral load. Patients of both cohorts were analyzed for EBV-specific T-cell responses by flow cytometry. All subgroups of patients were randomly selected but were representative for the respective cohort 1 or 2 in age, disease score and duration.

**Table 1:** Patient characteristics.

	Cohort 1	Cohort 2
n	63	387
Age, mean, range	47, 27–63	45, 20–78
Women, n (%)	46 (73)	245 (63)
Bell score, mean, range	30, 10–50	30, 10–70
Disease duration in years, mean, range	7.4<1–30	7.4, <1–39
History of autoimmune disease (%)	11.1*	9.3#
Deficiency of IgG/IgA/IgM (%)	4.4/3.6/3.1	15.2/2.9/0
Deficiency of IgG_3_ (%)	8.18	16.7

Hashimoto thyreoiditis (n = 6), lichen sclerosus (n = 1);

^#^ Hashimoto thyreoiditis (n = 30), psoriasis (n = 3), colitis ulcerosa (n = 1), Morbus Bechterew (n = 2).

### Blood samples

Blood and serum was obtained from CFS patients and healthy subjects. Peripheral blood mononuclear cells (PBMCs) were isolated by density gradient centrifugation using Ficoll Hypaque and either cryopreserved for T-cell analysis or directly used in cell culture stimulation assay for memory B-cell analysis.

### Quantitative real-time PCR

Detection of EBV-DNA in PBMCs was done by nested PCR for EBER-1 with the following primers forward 5′-TCC CGG GTA CAA GTC CCG-3′ and reverse 5′-TGA CCG AAG ACG GCA GAA AG-3′ at 900 nM. Detection has been performed with probe FAM-5′-TGG TGA GGA CGG TGT CTG TGG TTG TGT T-3′-TAMRA (Eurofins MWG Operon, Ebersberg Germany) at 5 µM. Amplification data were analyzed by an ABI PRISM 7700 Sequence Detection System (PE Applied Biosystems, California, USA). Successful DNA isolation was verified by histone replication with the primers forward 5′-CCA GAG CGC AGC TAT CGG T-3′ at 900 nM and reverse 5′-CAC GTT TGG CAT GGA TAG CAC -3′ at 50 nM and the probe FAM - 5′-GCA AGT GAG GCC TAT CTG GTT GGC CTT T-3- TAMRA (Eurofins MWG Operon, Ebersberg Germany) at 5 µM. For BZLF-1 the following primers forward 5′-AAATTTAAGAGATCCTCGTGTAAAACATC-3′ and reverse 5′-CGCCTCCTGTTGAAGCAGAT-3′ at 30 pM were used. Detection has been performed with probe FAM-5′-ATAATGGAGTCAACATCCAGGCTTGGGC-3′-TAMRA (Eurofins MWG Operon, Ebersberg Germany) at 10 pM. For detection of BZLF-1 RNA, isolated total RNA was reverse transcribed according to the manufacture's instructions (Life Technologies, Darmstadt, Germany). EBER copies/µg DNA and BZLF-1 copies/µg cDNA were calculated in accordance to standard EBV-copies [60]. Results ≥35 copies/µg DNA/cDNA were regarded as positive.

### Enzyme-linked immunosorbent assay (ELISA)/Enzyme immunoassay (EIA)

EBNA-IgG, VCA-IgG and VCA-IgM were detected using an immuno chemiluminescence assay (CLIA, DiaSorin, S.p.A., Saluggia, Italy) according to the manufacturer's instructions. EIA was used to detect EBV EBNA-1-IgG at the Labor Berlin GmbH.

### ELISpot assay

Analysis of memory B cells was adapted from Crotty *et al.* [61]. PBMCs were stimulated unspecifically with Pokeweed mitogen (PWM) at 10 ng/ml (Sigma Aldrich, Schnelldorf, Germany), Staphylococcus aureus Cowan at 1∶10000 dilution (Merck, Darmstadt, Germany) and CpG at 6 µg/ml (InvivoGen, CA, USA) in RPMI 1640 (PAA Laboratories, Cölbe, Germany) supplemented with Penicillin/Streptomycin 100× and L-Glutamine at 2 mM and 10% FCS (both Biochrom, Berlin, Germany) and β-Mercaptoethanol at 50 µM (Merck, Darmstadt, Germany) for 7 days at 37°C in 5% CO_2_. For T-cell independent stimulation B cells from CFS patients were enriched with a RosetteSep CD3 depletion kit according to the manufacturer's instructions (Stemcell Technologies, Grenoble, France). 2.5×10^6^ B cells per well were kept in 1 ml IMDM (PAA Laboratories, Cölbe, Germany) with 10% heat-inactivated FCS (Valley Biomedical, Winchester, VA, USA), 5 µg/ml insulin/transferrin and 5 ng/ml selenium (all Sigma Aldrich, Schnelldorf, Germany), 1.25 µg/ml CpG (Invivogen, CA, USA), 300 U/ml IL-2 (Chiron-Behring, Liederbach, Germany), 12.5 ng/ml IL-10 (ImmunoTools, Friesoythe) and 500 ng/ml IL-21 (ImmunoTools, Friesoythe, Germany) and 0.5 µg/ml anti-CD40 monoclonal antibody (R&D Systems, MN, USA. Cells were cultured for 7 days at 37°C in 5% CO_2_. After stimulation, the cells were transferred at a concentration of 1×10^6^/100 µl into a 96-well multiscreen HTS-IP filter plate (Merck Millipore, MA, USA) pre-coated with purified, recombinant EBV-VCA at 0.1 µg/well (tebu-bio, Le-Perray-en-Yvelines, France) and EBV-EBNA-1 at 1 µg/well (tebu-bio, Le-Perray-en-Yvelines, France) and purified EBV-lysate at 1∶20 dilution (tebu-bio, Le-Perray-en-Yvelines, France). For the analysis of total IgG, anti-human IgG-Fc-fragment antibody (Jackson Immunoresearch, PA, USA) was coated at a concentration of 1.2 µg/well and cells were seeded at a concentration of 1.25×10^4^/100 µl, 6250/100 µl and 3125/100 µl for 6 h. Secreted IgGs were detected using an anti-human IgG, F(ab′)2 fragment coupled to Biotin at 1 µg/ml (Biosource, Life Technologies, Darmstadt, Germany) and Horseradish Peroxidase Avidin D at 5 ng/ml (Vector Laboratories, MI, USA). IgG spots were visualized by adding 3-Amino-9-ethylcarbazole (Sigma-Aldrich, Schnelldorf, Germany). Plates were scanned and spots enumerated on a CTL Immunoplate reader using Immunospot Academic software (Cellular Technology Ltd, OH, USA). Frequencies were expressed as the ratio of the mean number of antigen-specific spots and mean number of total IgG spots.

### Cytokine analysis

Antigen-specific T-cell response was measured by cytokine production in cell culture supernatants of PBMCs stimulated with either 1 µg/ml SEB (Sigma-Aldrich, Schnelldorf, Germany), 1 µg/ml EBV total lysate or 1 µg/ml of the EBV peptide EBNA-1 (JPT, Berlin, Germany) for 48 h. 2×10^6^ PBMCs were kept in 1 ml serumfree RPMI (PAA Laboratories, Cölbe, Germany) with 2% Hepes buffer, 1% L-glutamin (Biochrom, Berlin, Deutschland) and 0.5% gentamycin (Merck, Darmstadt, Germany). IFN-γ, IL-10, IL-2 and TNF-α were measured in cell culture supernatants with a MPXHCYTO-60K Multiplex-Immunoassay (Merck Millipore, MA, USA) on a Luminex® 200™ (Luminex, TX, USA) according to manufacturer's instructions.

### T-cell expansion

EBV-specific memory T cells were analyzed after stimulation with EBNA-1 or CMV-pp65 peptides and expansion *in vitro* as recently described [62]. After overnight incubation of PBMCs in IMDM (PAA Laboratories, Cölbe, Germany) containing 10% AB serum (Valley Biomedical, Winchester, VA, USA) and supplemented with Penicillin/Streptomycin 100× and L-glutamine at 2 mM (both Biochrom, Berlin, Germany) at 37°C in 5% CO_2_, in 96-well round bottom plates at a concentration of 2×10^5^ cells per well with 50 IU/mL rhIL-2 (Chiron-Behring, Liederbach, Germany) and 10 ng/mL IL-7 (ImmunoTools, Friesoythe, Germany). On day 3, 5 and 7 media and IL-2 at 50 ng/µl were renewed. IL-7 at 5 ng/µl was added on day 7 of culture, and cells were harvested, washed and stained for cytokines.

### Multiparameter flow cytometry

Intracellular and extracellular staining was applied for T-cell analysis after 10 days of expansion. 2×10^6^ PBMCs were restimulated with an EBNA-1 or CMV-pp65 peptide pool (JPT, Berlin, Germany) at (1 µg/mL) or DMSO (Sigma Aldrich, Schnelldorf, Germany) as negative control for 5 h. Brefeldin A (7.5 µg/mL) (Sigma Aldrich, Schnelldorf, Germany) was added after 1 h of stimulation. Live/dead cells were discriminated using an amine reactive dye (Invitrogen, Life Technologies, Darmstadt, Germany) and stained with fluorescence conjugated monoclonal antibodies against CD3, CD4, CD8, PD-1, IFN-y, TNF-α and IL-2 (BD Biosciences, NJ, USA). Background events in DMSO controls were subtracted from events counted in response to EBNA-1 or CMV-pp65 stimulation. Data acquisition was performed on BD LSR II (Becton Dickinson, NJ, USA) and analysis was done using FlowJo software.

### Statistical analysis

Statistical data analysis was done using the software SPSS Statistics 19 and GraphPad Prism 5. Nonparametric statistical methods were used. Continuous variables were expressed as median and interquartile range (IQR), if not indicated otherwise. Univariate comparisons of two independent groups were done using the Mann-Whitney-U test. For association analysis Fisher's exact test was used. A p-value of <0.05 was considered statistically significant.

## Results

### A significant subset of CFS patients shows abnormal EBV serology

**Figure 1:**
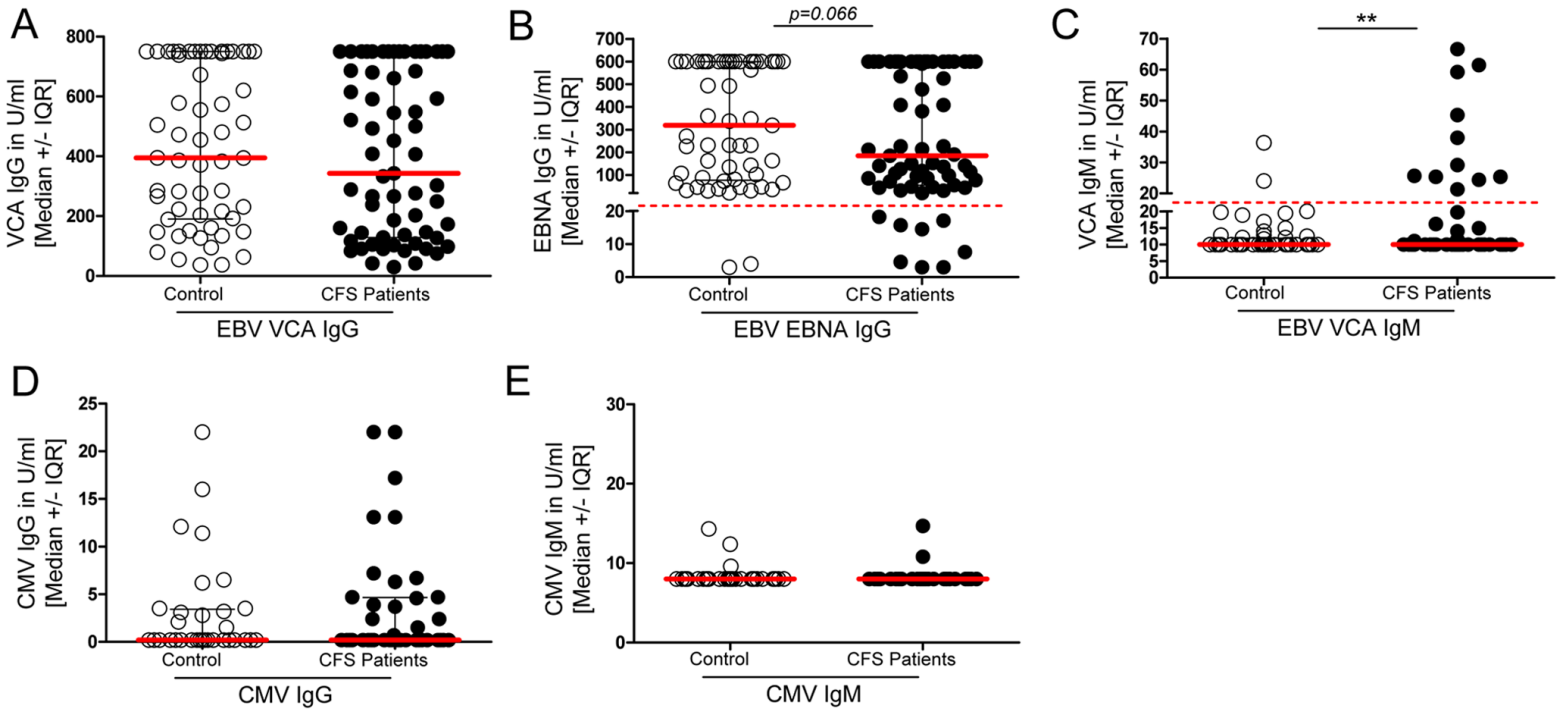
EBNA antibody response is reduced in CFS patients. (A) Serum IgG titers were assessed for healthy controls and CFS patients by ELISA for EBV VCA-IgG (control n = 57, CFS n = 63), (B) EBNA-IgG (control n = 57, CFS n = 63), (C) EBV VCA-IgM (control n = 57, CFS n = 63), (D) CMV-IgG (control n = 32, CFS n = 41) and (E) CMV-IgM (control n = 32, CFS n = 41). Statistical analysis was performed using the two-tailed Mann-Whitney-U test and for EBNA-IgG and EBV VCA-IgM Fisher's exact one-tailed test for association analysis with * p<0.05.

First, we analyzed the EBV-specific antibody response from CFS patients. We compared serum EBV-VCA-IgG, -IgM, and EBNA -IgG from patients of cohort 1 (n = 63, Table 1) and healthy controls (n = 57) (Figure 1A). While we did not observe a difference in levels of VCA-IgG, IgG antibodies against EBNA were undetectable (≤20 U/ml) in 12.7% of CFS patients in contrast to 3.5% of healthy controls (p = 0.06, Figure 1B). When excluding the values which were out of range, we obtained similar results with comparable levels for VCA-IgG and a significant difference for EBNA-IgG (p = 0.05, data not shown).

Further, elevated VCA-IgM was found more frequently in patients compared to healthy controls (17.5 vs. 3.5% p = 0.013, Figure 1C). In contrast, CMV-specific IgM was elevated in only 2 patients and levels of IgG revealed no difference between CFS patients and healthy controls (Figure 1D and Figure 1E). Within this cohort of patients only one patient with positive EBV-VCA IgM showed lack of EBNA-IgG. Thus, an abnormal EBV serology (positive VCA-IgM or negative EBNA-1-IgG) was detected in 30% of CFS patients.

**Figure 2:**
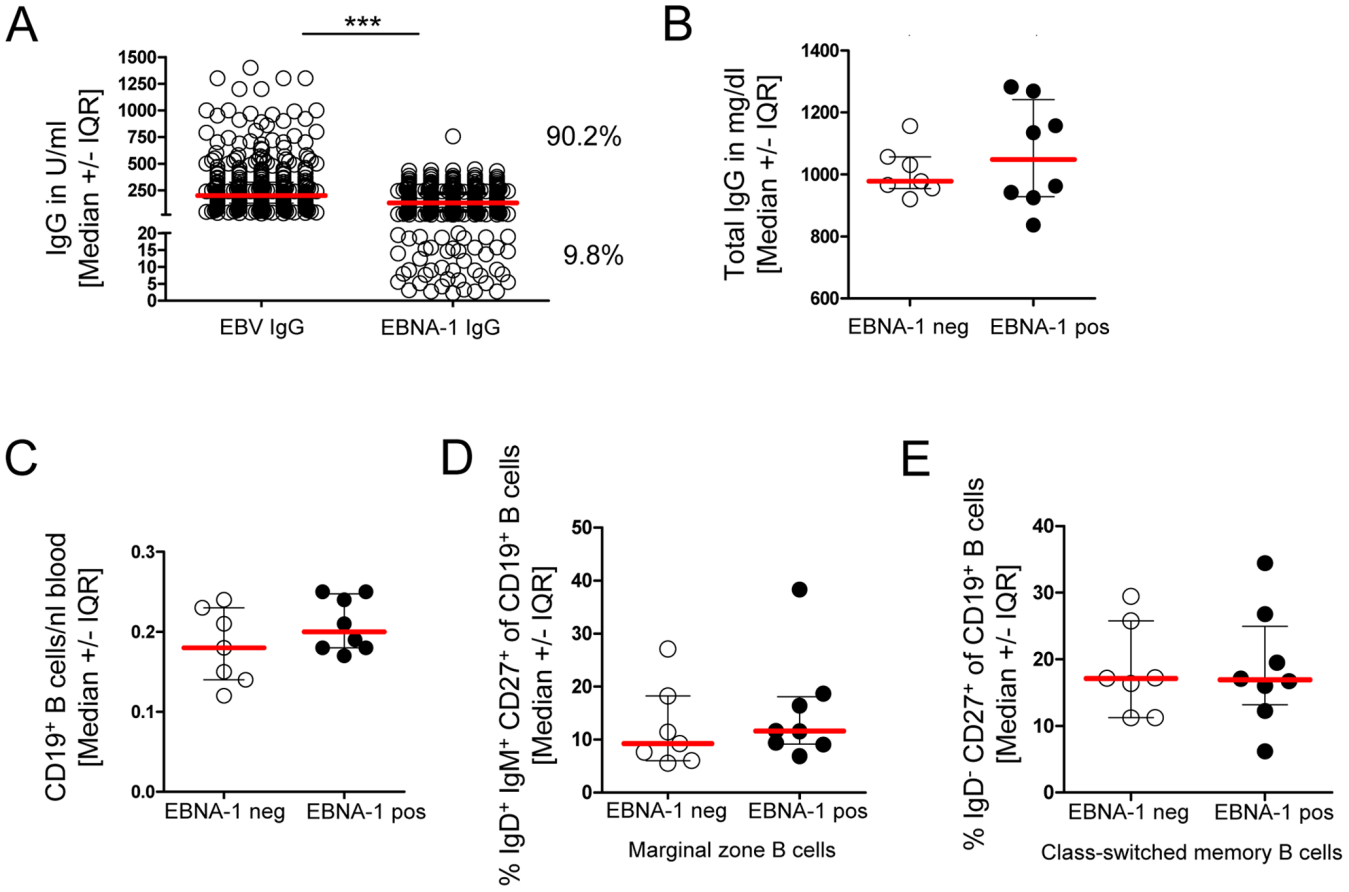
EBNA-1-IgG is reduced in a subset of patients but total IgG and B-cell subpopulations are not different in EBNA-1-IgG positive and -negative CFS patients. (A) Serum IgG titers were assessed in CFS patients for EBV-IgG and EBV-EBNA-1-IgG (n = 387), (B–E) EBNA-1 negative (neg, n = 7) and positive (pos, n = 8) CFS patients were compared for (B) total IgG, (C) the absolute numbers of CD19^+^ B cells/nl blood, (D) frequencies of IgD^+^IgM^+^CD27^+^ marginal zone B cells, and (E) frequencies of IgD^−^CD27^+^ class switched memory B cells. Statistical analysis was performed using the two-tailed Mann-Whitney-U test and for IgG Fisher's exact one-tailed test for association analysis with *** p<0.0001.

In a second consecutive cohort with 387 CFS patients (Table 1), EBV-specific antibodies were measured by ELISAs that determine IgG against a mixture of various EBV proteins or EBNA-1, respectively. Similarly, we observed a lack of EBNA-1-IgG in 9.8% of EBV-IgG positive patients (Figure 2A). In a randomly selected subset of 8 EBNA-1-IgG positive and 7 negative patients we further comparatively analyzed total IgG levels, frequencies of B cells, and B-cell subsets. No difference in total IgG (Figure 2B) was found in EBNA-1-IgG negative compared to EBNA-1-IgG positive CFS patients. Moreover, the absolute B-cell numbers (Figure 2C) and frequencies of memory B-cell subsets were not different among EBNA-1 negative and positive patients (Figure 2D and Figure 2E) and were within the normal range compared to the reference values of our immune diagnostic laboratory.

### EBV-specific memory B cells are low or absent in most CFS patients

**Figure 3:**
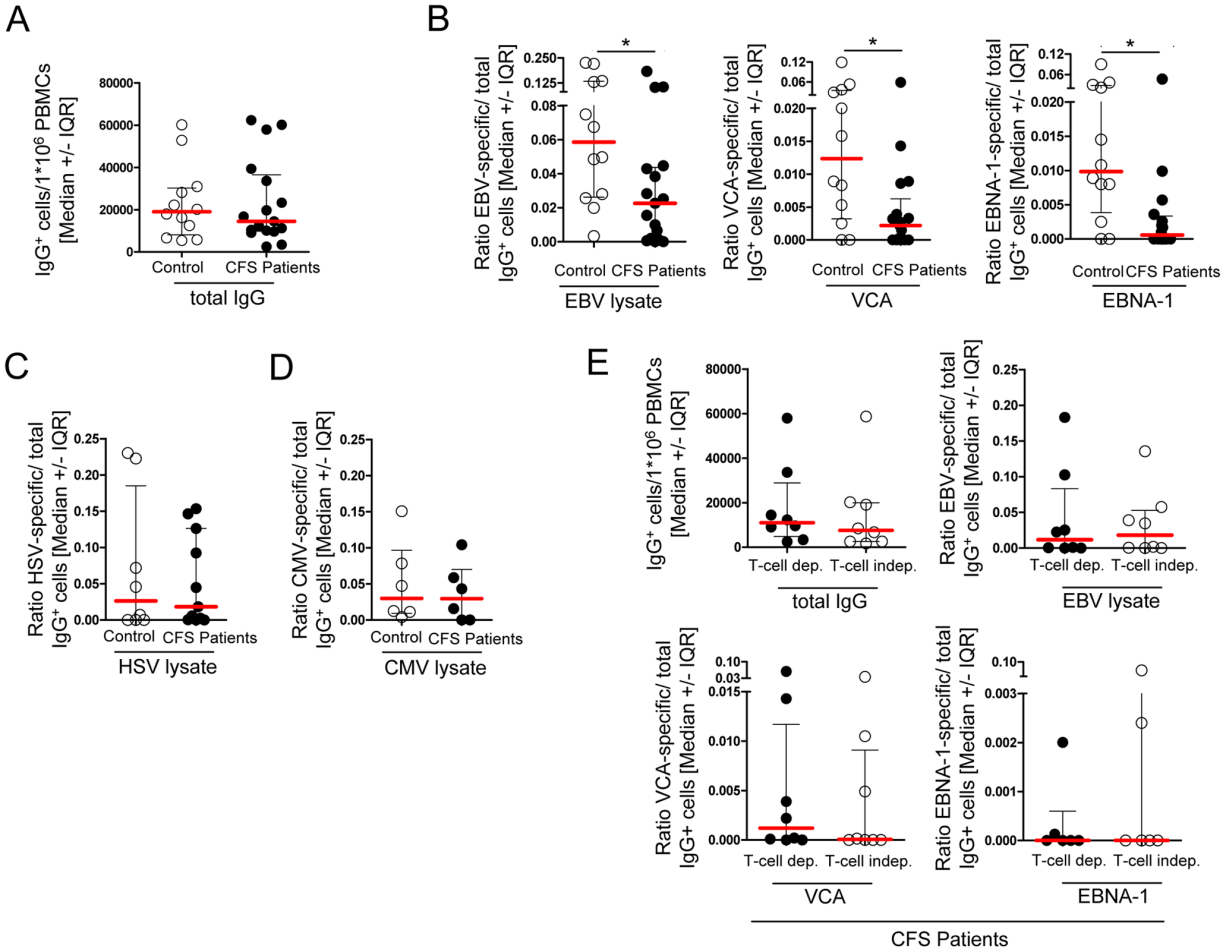
EBV-specific antibody secreting cells are reduced in CFS patients. (A–D) Frequencies of ASCs in healthy controls and CFS patients 7 days after polyclonal stimulation of total PBMCs. Secreted total and specific IgG was assessed with the ELISpot assay. IgG-secreting B cells are shown as frequencies from 1×10^6^ seeded cells for (A) total IgG (control n = 12, CFS n = 17), (B) EBV-lysate-specific IgG (control n = 12, CFS n = 17), VCA-specific IgG (control n = 12, CFS n = 17), IgG against EBNA-1 peptides (control n = 12, CFS n = 16) and (C) HSV- (control n = 8, CFS n = 11) and (D) CMV-lysate-specific IgG (control n = 6, CFS n = 6). (E) Comparison of frequencies of ASCs in polyclonal stimulation of total PBMCs (T-cell dependent) and stimulation of isolated B cells with CD40L (T-cell independent) in CFS patients for total IgG, EBV-lysate, (n = 8), VCA or EBNA-1 peptides (n = 6). Statistical analysis was performed using the two-tailed Mann-Whitney-U test with * p<0.05.

Serum IgG antibodies are mostly derived from long-living plasma cells, which have settled in the bone marrow, often many years before. Thus, disturbance of memory B-cells can be overlooked for long time if only IgG serum levels are analyzed. Therefore, we studied the frequencies of specific antibody-secreting cells (ASCs) derived from EBV-specific memory B cells after *in vitro* restimulation by activating PBMCs with a mixture of CpG, SAC, and PWM for 7 days. ASCs were analyzed in the ELISpot assay. No difference between CFS patients and healthy controls was detected for total IgG secreting memory B cells (Figure 3A). However, the CFS patients had significantly reduced frequencies of B cells producing antibodies against EBV antigen mixture (EBV-lysate). In addition, by using overlapping peptide pools, VCA-specific and EBNA-1-IgG secreting B cells were analyzed. A diminished B-cell memory response was defined by frequencies below the interquartile range of the control group. As shown in Figure 3B a diminished B-cell memory response against EBV-VCA was observed in 59% of CFS patients, and against EBV-EBNA in 76% of patients, respectively. We found no correlation between frequencies of VCA- or EBNA-specific memory B cells and levels of IgG antibodies (VCA r = −0.1242, p = 0.67 and EBNA r = 0.07913, p = 0.8). Patients analyzed for memory B cells were derived from cohort 1 and their IgG titers were comparable to the IgG titers of the whole cohort 1 (median VCA-IgG 350 vs. 450 U/ml, EBNA-IgG 100 vs. 180 U/ml, shown in Figure 1B). We determined HSV- and CMV lysate-specific ASCs in a subgroup of these patients detecting no difference between patients and controls further excluding a general B-cell defect (Figure 3C and Figure 3D). To assess whether the reduction of EBV-specific memory B cells is possibly due to the presence of suppressive T cells in culture, enriched B cells were cultivated without T cells in the presence of feeder LL8 cells. Soluble CD40 ligand together with a cytokine mix and CpG were used to induce memory B-cell differentiation. Similar numbers of EBV-specific antibody secreting B cells were detected in patients in the T-cell dependent and independent analysis (Figure 3E). Taken together, the lack or reduced level of memory B cells shows a more profound deficiency of the EBV-specific B-cell response as evident from serology.

### CFS patients show diminished T-cell cytokine response to EBV

**Figure 4:**
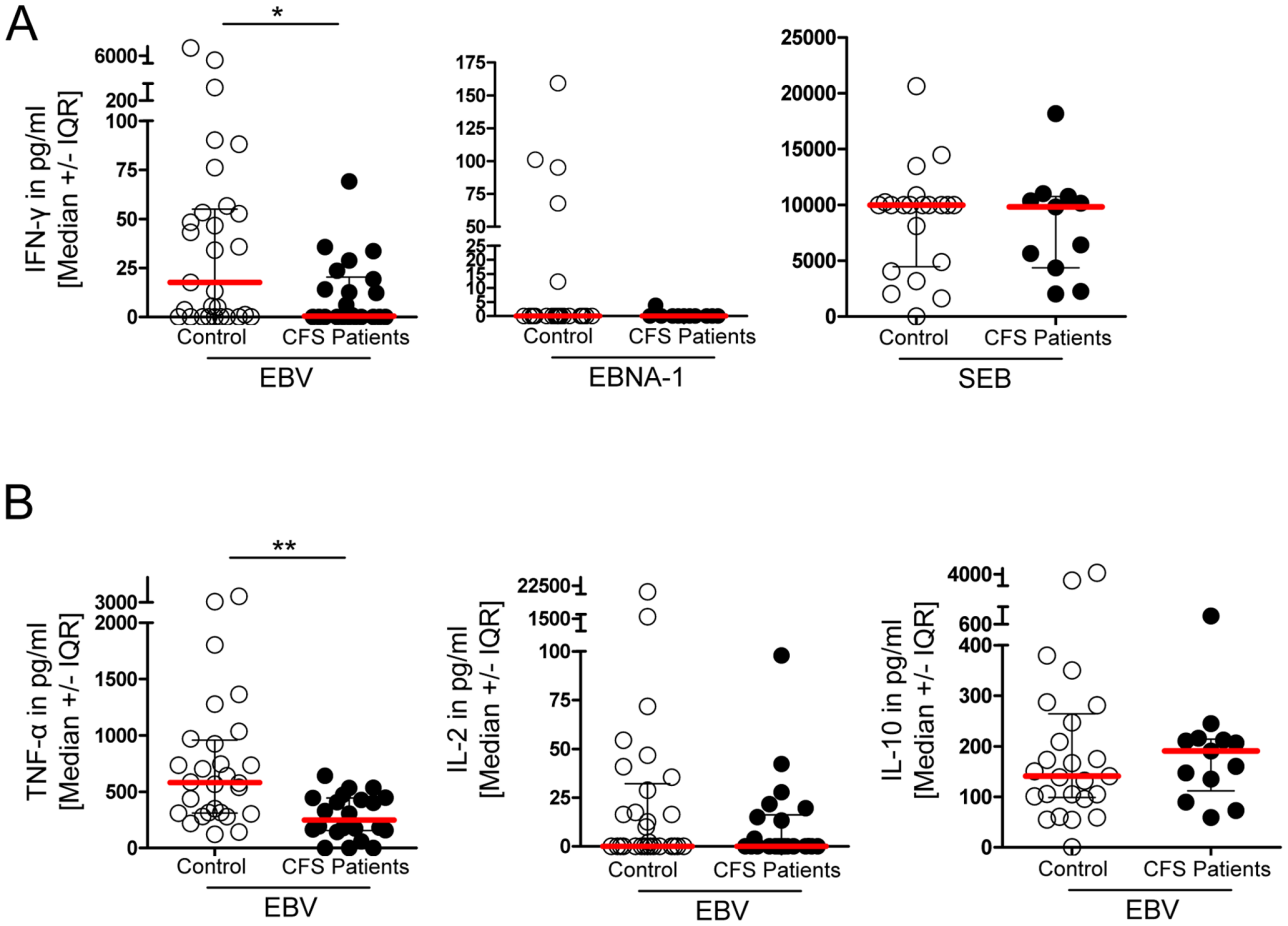
CFS patients show diminished cytokine response against EBV. Whole blood of healthy controls and CFS patients was analyzed by Multiplex-Immunoassay for (A) IFN-γ production after stimulation with either EBV-lysate (control n = 29, CFS n = 22), EBNA-1 peptide (control n = 24, CFS n = 11) or SEB (control n = 21, CFS n = 11) and (B) after EBV-lysate stimulation for TNF-α (control n = 29, CFS n = 22), IL-2 (control n = 29, CFS n = 22) and IL-10 (control n = 25, CFS n = 13). Statistical analysis was performed using the two-tailed Mann-Whitney-U test with * p<0.05 and *** p<0.001.

In a next series of experiments, we analyzed the EBV-specific T-cell response in patients and healthy controls. First, EBV-lysate induced production of several cytokines that were tested in whole blood, revealed a significantly reduced number of IFN-γ responders in the patient group with 50% (n = 11/22) vs. 69% (n = 20/29) in the control group (Figure 4A). Using whole protein-spanning overlapping 15-mer peptides from EBNA-1 protein for stimulation, no patient showed a detectable IFN-γ response (Figure 4A). Similar IFN-γ levels were observed in response to the T-cell superantigen SEB in patients and controls. In addition, a significant reduction of TNF-α and a lower number of patients producing IL-2 was observed in response to EBV-lysate, while the IL-10 response was not diminished (Figure 4B).

### CFS patients show reduced EBV-specific multifunctional memory T cells

**Figure 5:**
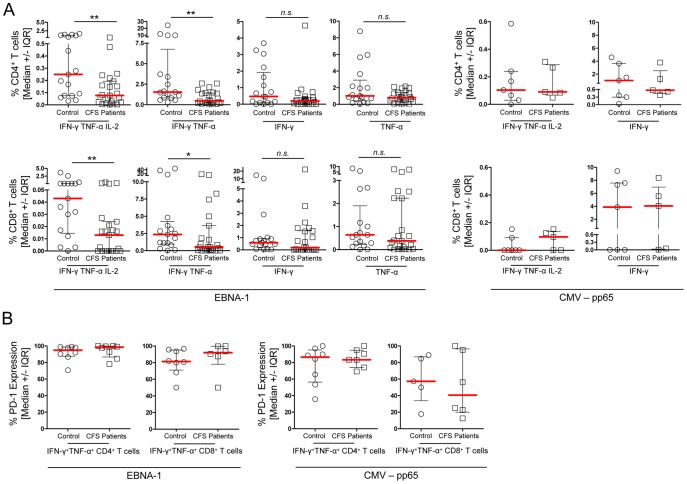
CFS patients show reduced EBV-specific memory T-cell response. (A) Comparison of cytokine producing CD4^+^ (upper panels) and CD8^+^ T cells (lower panels) of CFS patients and healthy controls after 10 days of stimulation with EBNA-1 (left panel, Control n = 17, CFS n = 23). Boolean gating strategy was applied to analyze IFN-γ/TNF-α/IL-2 triple, IFN-γ/TNF-α double, and IFN-γ and TNF-α single cytokine producing T cells after intracellular staining of isolated PBMCs incubated with Brefeldin A for 16 h. Stimulation with CMV pp65 (right panel, Control n = 7, CFS n = 5) is shown for IFN-γ/TNF-α/IL-2 triple, and IFN-γ single cytokine producing T cells. (B) Frequencies of PD-1 expression were analyzed for IFN-γ/TNF-α double producing CD4^+^ and CD8^+^ T cells after 10 days of stimulation with EBNA-1 or pp65 (n = 8). Statistical analysis was performed using the two-tailed Mann-Whitney-U test with ** p<0.01.

To analyze the EBV-specific memory T-cell response in more detail, we stimulated PBMCs with EBNA-1 peptides and expanded them *in vitro* in the presence of IL-2 and IL-7 for 10 days as recently described [60]. IFN-γ^+^ TNF-α^+^ and IL-2^+^ producing CD3^+^CD4^+^ and CD3^+^CD8^+^ T cells were analyzed by flow cytometry. Frequencies of EBV-specific T cells were lower in CFS patients (n = 23) compared to the control group (n = 17, Figure 5A). The difference was most evident in the IFN-γ^+^ TNF-α^+^ IL-2^+^ triple producing - so called multifunctional - CD4^+^ and CD8^+^ T cells. In addition, the frequencies of the IFN-γ^+^ TNF-α^+^ double producers were significantly lower in the patient group. No difference was observed for the single producers. A diminished EBV-specific T-cell response was defined by frequencies below the interquartile range of the control group in CD4^+^ and CD8^+^ triple cytokine producing T cells. Based on this definition a diminished response was detected in 48% of CFS patients for triple CD4^+^ T cells and in 52% of patients for triple CD8^+^ T cells. As control, we analyzed the T-cell response against CMV peptide pp65 in a subset of CMV-reactive subjects (Figure 5A, right panel) showing no difference among triple and double CMV-reactive T cells between patients (n = 5) and controls (n = 7). Comparing the total cell numbers of cultures stimulated with EBNA-1, no difference was observed (patients median 77%, range 44%–106% of initial cell number, controls median 93%, range 28%–148% of initial cell number). Next, we analyzed the expression of the exhaustion marker PD-1 on EBNA-1- and CMV-pp65-specific T cells but found no difference in the frequencies of PD-1 expression between patients and controls as shown for the population of the IFN-γ^+^ TNF-α^+^ double producing CD4^+^ and CD8^+^ T cells in Figure 5B (n = 8).

### Evidence of enhanced latent EBV replication in CFS patients

**Figure 6:**
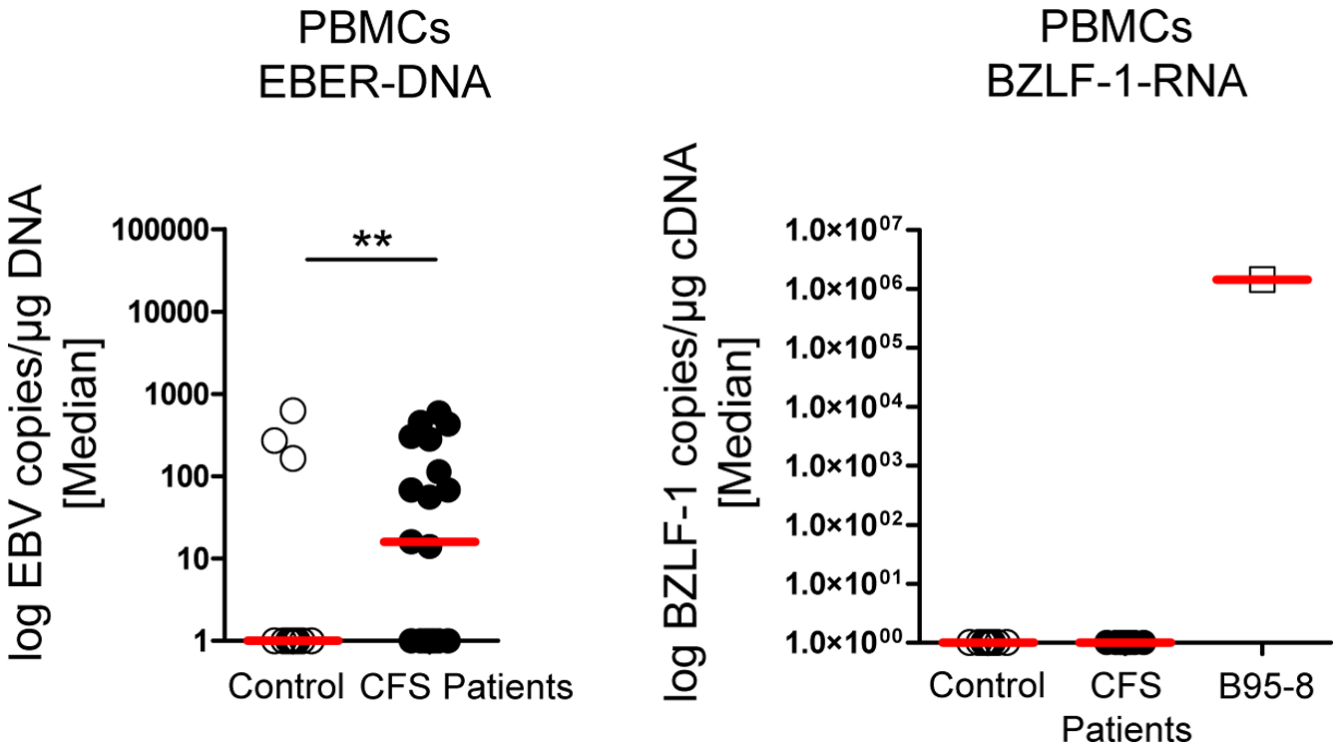
Latent EBV can be detected more frequently in CFS patients. (A) EBV DNA was analyzed via nested real-time PCR in total PBMCs of 20 healthy donors and CFS patients for EBER-1. EBER-copies were calculated in accordance to Namalwa standard. (B) BZLF-1 RNA was analyzed via nested real-time PCR in total PBMCs of 20 healthy donors and CFS patients but no BZLF-1 cDNA was detected. cDNA of EBV cell line 293T/B95-8 was used as positive control. Statistical analysis was performed using the one-tailed Mann-Whitney-U test with ** p<0.01.

As a measure of the EBV load, we determined EBER DNA by real-time PCR in whole blood. EBER-DNA was detectable in 21 of 290 patients (7.2%) in a low copy number (<1000 – 2.930 copies/ml). In a subset of patients and controls we comparatively analyzed EBV DNA in isolated PBMCs. EBER-DNA was detectable in 55% of patients (n = 11/20) compared to 15% (n = 3/20) of healthy controls (p<0.01) (Figure 6A). No EBER-DNA was detected in the plasma (data not shown). Additionally, we tested 4 EBV seronegative CFS patients and detected no EBER DNA in PBMCs (data not shown). As a marker for lytic replication we further analyzed RNA levels of the lytic protein BZLF-1 in PBMCs (n = 20) but could not detect BZLF-1 cDNA in patients or controls (Figure 6B). As positive control copies/µg cDNA of EBV cell line 293T/B95-8 were determined.

## Discussion

For many years, researchers have suspected EBV to be involved in CFS. A hallmark of CFS is chronic activation of the immune system, which can be triggered by infections or non-infectious agents [63], [64], [65]. Although altered EBV-specific antibody titers have been repeatedly demonstrated in CFS, no clear evidence for chronic EBV replication has been obtained so far. To contribute to the understanding of CFS, our present study aims to further elucidate the immune response to EBV in CFS.

First of all we could confirm previous reports providing serological evidence of EBV reactivation by demonstrating elevated IgM antibodies against the late VCA antigen in a subset of patients [39], [31], [41]. Remarkably, in line with this finding we could provide evidence of enhanced viral load of EBV by detection of EBV DNA in a significantly higher proportion of patients compared to healthy controls. However, no patient showed a high viral load as seen in EBV-associated post-transplant lymphoproliferative disorder or acute infectious mononucleosis. Further, we had no evidence of lytic replication as we could neither detect EBER DNA in plasma nor BZLF-1 RNA in PBMCs. Thus, our findings suggest a higher level of latency-associated replication in CFS patients.

A key finding in our study was the lack of IgG antibodies to EBNA-1 antigen, observed in a subset of patients in 2 different cohorts. EBNA-1 is one of the few gene products expressed in type I latency. Assessment of EBNA-IgG is used for diagnosis of EBV infection, as it is mounted rather late during primary infection and a failure to produce EBNA-IgG had been observed both in severe infectious mononucleosis and chronic active EBV disease [66], [67]. However, serum IgG levels do not reflect the number of B-cell memory cells as serum IgG is derived from long-lived plasma cells, which often have been acquired many years ago. To investigate the memory B-cell response in more detail, we analyzed the *in vitro* differentiated EBV-specific memory B-cell pool revealing a much more profound defect in EBV-directed B-cell response with low or undetectable EBV-specific ASCs in the majority of patients. Remarkably, memory B-cell responses not only against EBNA-1, but also against the late lytic antigen VCA were low to absent in the majority of patients despite normal IgG-VCA titers indicating a secondary exhaustion of the memory B-cell pool. Alternatively, an impaired ability to mount a sufficient number of EBV-specific memory B cells upon primary infection may be discussed. This deficiency seems to be specific for EBV as total IgG, CMV-specific antibodies and CMV- and HSV-specific B-cell memory responses were not different compared to healthy controls. Further, no differences in the amount of total IgG or B cells and memory B cells was observed between EBNA-1-IgG negative and -positive patients. The failure to detect ASCs *in vitro* might either be related to a loss of memory B cells or their failure to differentiate into ASCs. To exclude that T cells in CFS patients inhibit or kill *in vitro* differentiating EBV-memory B cells, we additionally used a T-cell independent ASCs-inducing protocol showing similar results.

In accordance with the diminished EBV-specific memory B-cell response, we could demonstrate an impaired EBV-specific T-cell response, particularly of the triple and double cytokine producers. Based on our findings we assume frequent EBV reactivation as a mechanism for the impaired EBV-specific B- and T-cell immune responses in CFS patients. An impairment of specific T-cell responses is observed in various chronic infections. In HIV and HCV infection diminished specific polyfunctional CD8^+^ T cells were described [68], [69], [70], [71]. Further, selective lack of polyfunctional T cells was demonstrated in animal models of chronic SIV and Leishmania infection accompanied by the absence of circulating antibodies [72], [73]. The persistence and continuous exposure to antigen may drive T cells into exhaustion. This process is often accompanied by the presence of high levels of IL-10 and upregulation of immune suppressor molecules [74].

Our findings in CFS have similarities to recent studies in systemic lupus erythematosus (SLE) as well, in which EBV reactivation is thought to play an important role in disease pathogenesis [75]. It is thought that the increased viral load or an altered presentation of EBV proteins that cross-react with cellular antigens may trigger pathogenic processes through molecular mimicry [56], [76], [77], [78], [79]. Abnormal expression of both viral lytic genes and impaired EBV-specific T-cell responses were demonstrated in SLE patients [80], [81]. However, in contrast to our findings in CFS, increased levels of EBV/EBNA-1 directed antibodies were reported for SLE as well as for patients with multiple sclerosis [80], [82]. Further EBV-specific polyfunctional T cells were shown to have higher levels of PD-1 in SLE compared to healthy controls [81], a finding we did not observe in our patients.

Finally, we think the altered pattern of the specific immune response to EBV may be suitable as a diagnostic marker for CFS. The most prominent finding is the very low or absent B-cell memory response to EBV in the majority of CFS compared to healthy controls. Therefore we are currently evaluating the antibody responses against a broad variety of EBV peptides from 8 different proteins by an EBV seroarray. Further, we are evaluating fluorescence-labelled EBNA- and VCA peptides for the *ex vivo* quantitation of specific memory B cells by flow cytometry.

Taken together, our study provides clear evidence that deficiency of EBV-specific immune response is present in CFS. As EBV is known to be controlled by cell-mediated immunity, a diminished memory T- and B-cell response may result in impaired control of EBV. EBV replication is risk factor for development of lymphomas and autoimmune diseases both occurring at enhanced frequencies in CFS patients [83], [84].
